# ArrayWiki: an enabling technology for sharing public microarray data repositories and meta-analyses

**DOI:** 10.1186/1471-2105-9-S6-S18

**Published:** 2008-05-28

**Authors:** Todd H Stokes, JT Torrance, Henry Li, May D Wang

**Affiliations:** 1 Department of Electrical and Computer Engineering, Georgia Institute of Technology, Van Leer Building, 777 Atlantic Drive NW, Atlanta, GA, 30332, USA; 2 Biomedical Engineering, Georgia Institute of Technology and Emory University, Whitaker Building, 313 Ferst Drive, Atlanta, GA, 30332, USA; 3 Hematology and Oncology, Winship Cancer Institute, Emory University, 1365C Clifton Road, Atlanta, GA, 30322, USA; 4 Parker H. Petit Institute for Bioengineering and Bioscience, Georgia Institute of Technology, 315 Ferst Drive, Atlanta, GA, 30332, USA

## Abstract

**Background:**

A survey of microarray databases reveals that most of the repository contents and data models are heterogeneous (i.e., data obtained from different chip manufacturers), and that the repositories provide only basic biological keywords linking to PubMed. As a result, it is difficult to find datasets using research context or analysis parameters information beyond a few keywords. For example, to reduce the "curse-of-dimension" problem in microarray analysis, the number of samples is often increased by merging array data from different datasets. Knowing chip data parameters such as pre-processing steps (e.g., normalization, artefact removal, etc), and knowing any previous biological validation of the dataset is essential due to the heterogeneity of the data. However, most of the microarray repositories do not have meta-data information in the first place, and do not have a a mechanism to add or insert this information. Thus, there is a critical need to create "intelligent" microarray repositories that (1) enable update of meta-data with the raw array data, and (2) provide standardized archiving protocols to minimize bias from the raw data sources.

**Results:**

To address the problems discussed, we have developed a community maintained system called ArrayWiki that unites disparate meta-data of microarray meta-experiments from multiple primary sources with four key features. First, ArrayWiki provides a user-friendly knowledge management interface in addition to a programmable interface using standards developed by Wikipedia. Second, ArrayWiki includes automated quality control processes (caCORRECT) and novel visualization methods (BioPNG, Gel Plots), which  provide extra information about data quality unavailable in other microarray repositories. Third, it provides a user-curation capability through the familiar Wiki interface. Fourth, ArrayWiki provides users with simple text-based searches across all experiment meta-data, and exposes data to search engine crawlers (Semantic Agents) such as Google to further enhance data discovery.

**Conclusions:**

Microarray data and meta information in ArrayWiki are distributed and visualized using a novel and compact data storage format, BioPNG. Also, they are open to the research community for curation, modification, and contribution. By making a small investment of time to learn the syntax and structure common to all sites running MediaWiki software, domain scientists and practioners can all contribute to make better use of microarray technologies in research and medical practices. ArrayWiki is available at .

## Background

ArrayWiki improves on existing microarray repositories by providing an open interface for community curation (either through human or automated means), variance heatmaps and quality scores for every imported chip data file, and a convenient and compact data transport format that allows for storing data at a higher detail level and also provides visual assurance that the provided data is complete and is not corrupt.

### Microarray analysis and reproducibility

Microarrays are widely used to discover new markers of disease, to validate results of genetic engineering, and to evaluate toxicity of therapeutics [1-4]. The United States Food and Drug Administration (FDA) recently completed a large-scale evaluation of microarray data quality [5]. This was the first step in developing policies for microarray evidence in clinical trial documentation for new drugs. Although microarrays have been criticized for low reproducibility [6], recent findings of the FDA Microarray Quality Control (MAQC) consortium indicate that microarrays are in fact reproducible and consistent across different labs [7].

However, there still exist several problems. First, there are no current standards in microarray data analysis. Long-trusted standards (GCRMA, RMA, MAS5.0) for performing gene calculations [8,9] on the industry's most popular chip (Affymetrix) were bested by PLIER [10] in the FDA's titration tests. The *de facto *standard library for microarray data analysis is Bioconductor [11], which is an open-source project built on the R Programming Environment. Bioconductor contains many commonly-used routines for extracting, normalizing, visualizing and converting Affymetrix data files to gene expression values. These methods, however, use statistical modelling and redundant information rather than explicit data about probe (base pair) affinity to transform probe intensity data into gene expression estimates.

**Figure 1:**
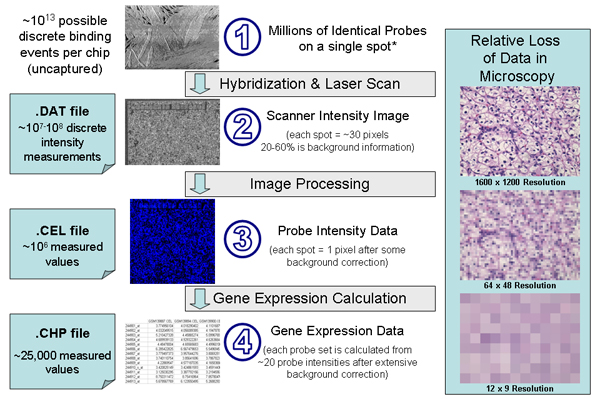
**Diagrams showing the loss of data and precision during microarray processing**. * Electron microscopy image of a microarray (adapted from reference [39]). In [20], the authors predict that 90% of CEL files generated from microarray experiments have never been deposited to any repository. Our own searches find only 6 experiments in GEO with associated DAT files.

Second, the motivation for archiving microarray data is to avoid duplicating experimental efforts, but this purpose is defeated if the data analysis methods are outdated and the source data is not available to reprocess using new algorithms. For example, when databases store only gene expression information without the underlying intensity measurements (e.g. GEO's SOFT file), any data produced by MAS 5.0 immediately becomes outdated when Affymetrix GCOS and PLIER become available. Comparisons between experiments where different gene calculation methods are used are suspect. Figure 1 illustrates the importance of archiving data at the maximum level of detail (scanner intensities). These files are much larger than those maintained by existing repositories and are virtually impossible to find from public sources. As analysis algorithms improve, ArrayWiki experimental results can be updated by a community effort to help the users.

### Survey of public microarray repositories

This paper is the result of studying the strengths and weaknesses of current microarray data repositories. These include Gene Expression Omnibus (GEO) [12], ArrayExpress (AE) [13], caArray Database (caA) [14], Stanford Microarray Database (SMD) [15], and oncoMine (OM) [16,17]. This is only a small portion of the many gene expression repositories, and does not include those for small research communities based on interest (BarleyBase [18]) or affiliation (University of North Carolina Microarray DB [19]).

More recently, a group from University of California, Los Angeles published Celsius [20], an effort to merge all Affymetrix data from disparate repositories into one location, available through a single programmatic interface. The authors support the importance of this work for three main reasons: the microarray repository field became very fragmented, data at the CEL file level is difficult to find even in the largest repositories, and experiments are annotated inconsistently across repositories.

All of these databases represent important efforts for ensuring that resources spent on microarray experiments are not lost or hoarded, but are preserved for future generations of researchers, bioinformaticians and wet lab experimentalists. However, most of these databases fail to provide any chip quality information. Also, they do not offer a familiar Wiki interface for data curation. Celcius is the only other repository intended to support community-driven meta-data curation, but that functionality is only available through a clumsy programmatic interface. Finally, none of these repositories have made a noticeable effort to include the Affymetrix DAT file type in their experiment records. The DAT files available in ArrayWiki offer the highest possible detail level about public experiments and allow bioinformaticians to improve on the algorithms used by Affymetrix software.

**Figure 2:**
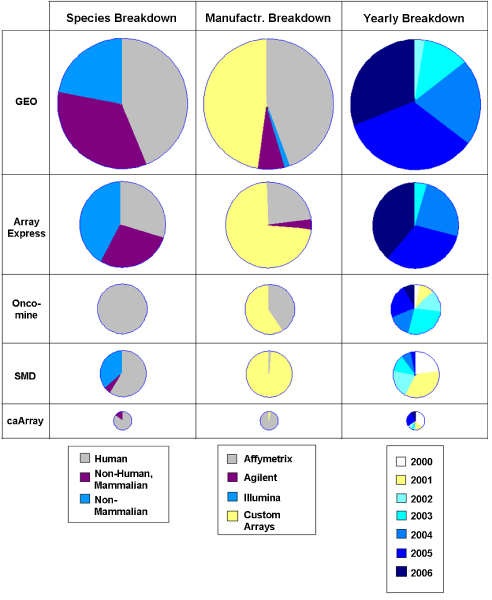
**Comparison of microarray repository contents**. The relative size of each pie corresponds to the number of experiments contained in each repository. Key observations include that SMD does not contain much recent data. One data artifact is found in the caArray Yearly Breakdown. An abnormal number of experiments show a date of '1-1-2000' because that is the default value in the data entry form and validation of the data is inadequate.

Figure 2 summarizes the contents of the archives being studied in our work.

The measured values in Figure 2 represent classifications of microarray chips as reported by the repository source and confirmed by our efforts. In general, GEO seems to be dominating the field due to its ability to recruit data uploads. As a result, it also contains the most current data. Another dominant technology is the Affymetrix platform. A combination of user-friendly lab equipment and simple data and protocol standards have led to the widespread use of their chip platforms. The oncoMine web site is a compendia rather than a repository. This means that rather than aiming to collect vast quantities of data, its goal is to collect only data of the highest quality for analysis of a specific disease: cancer. caARRAY is also a cancer-related database without the extra quality requirement of oncoMine (which is not considered beyond Figure 2).

**Figure 3:**
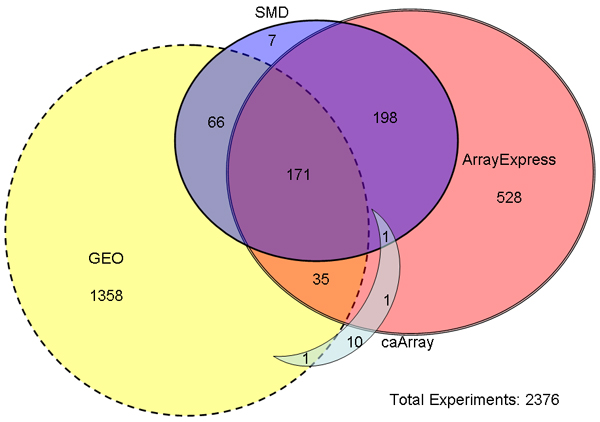
**Venn diagram showing overlaps in experimental data between repositories**. In [20], a similar Venn diagram is given representing all data collected in the effort to build the repository. This diagram differs in that it is not limited to Affymetrix experiments with available CEL files. This may account for the discrepancy in overlap between GEO and ArrayExpress in the two results.

Figure 3 depicts the overlap in experiments between four popular repositories. A standard procedure was used to generate this figure. All datasets examined were public, and had submission (or release) dates between August 2005 and June 2006 inclusive. Each dataset was searched in every other database with no date criteria. The criteria for determining matching datasets were species, platforms, authors, affiliation and publication (if available). This was repeated for each database. Our interpretation of Figure 3 is that repositories developed for different communities have become "silo-ed" over time, meaning that the contributors have not been sharing experiment records among the different repositories to make them more complete. This finding is in agreement with the authors of Celsius. The majority of experiments are found in only one repository (1358 + 528 + 10 + 7 = 1903 or 80%). Experimenters tend to patronize a particular repository, and the only evidence of an effort to merge repositories with the purpose of facilitating large-scale data mining is the incorporation of SMD experiments into ArrayExpress and GEO at certain points. This means that bioinformatics researchers must search all repositories to ensure they've collected all public data relevant to a particular topic.

Finally, despite the cost of obtaining tissue samples and the complexity of analysis and interpretation, human and mammalian chips still outnumber all other sample organisms (e.g., cell lines, plants, and single-celled organisms). This statistic may be inflated by the failure of many repositories to distinguish between samples taken directly from human tissues and those from genetically modified human cell lines.

### Usability of existing repository interfaces

Designing easy-to-use and clean interfaces to assist *data providers *and *data consumers *to *upload *and *download *is critical for expanding the reach of microarray repositories. However, the usability of existing repositories is hampered by a lack of shared standards for minimally required experimental data.

First, existing repositories have different requirements for data submission, and vary in their degree of openness to community involvement. These repositories vary in their ease of use of human interfaces (e.g., web sites), in the availability of programmatic access through Application Programming Interfaces (APIs) and their availability of data for bulk download (e.g., the entire database available through FTP or export of search results in XML format). In general, GEO and ArrayExpress have good web sites and APIs, but any effort to merge their datasets (as in ArrayWiki) requires developers to learn a variety of interfaces (Custom XML and SOFT files for GEO, MAGE-ML and seven file formats for ArrayExpress). Being the earliest developed repository, SMD does not make use of recent advances in usability such as JavaScript and AJAX. However, its functionality has been updated over time based on feedback from users and thus is far better than caArray, which is slow to respond and does not provide advanced search functionality.

Second, the existing repositories have different policies with regard to the timeline of making uploaded data available for public consumption. In some cases, this is a service to authors to allow them to use processing tools while keeping data private until publication. For example, GEO's express policy is to make data public automatically after six months.

Third, the existing repositories have different data verification and curation because the database administrators vary. Some repositories will exchange emails with individuals making submissions to check facts. Regardless of the intent many problems still make it into the final repository, including corrupt file formats and missing probe intensity files. Many experiment records claiming to include 200+ chips may only contain half as many files in the associated compressed probe data file.

Finally, the existing data repositories do not provide scanner intensity data (as DAT files for Affymetrix chips), even though this data is extremely useful for quality control procedures. The absence of this data type certainly confounds down-stream data analysis because the artifacts caused by instrument and experimental procedures can not be double-checked by the users.

### Data maintainability of existing repositories

Meta-data in existing repositories are usually problematic due to lack of standard in data maintainability design. The consumers of data, rather than the providers of data, are most likely to find problems in the meta-data. However, current repository structure prohibits data consumers from modifying the source records. These researchers must contact the original source of the data using the provided email, which may no longer be valid.

One category of problem is the lack of meta-data. Most of the repository query interfaces are optimized for finding specific experiments from the literature, which is the first step taken by clinicians or biologists. (Based on the comparison survey we conducted, connections between experiments and PubMed are usually accurate.) However, they often do not provide technical features, i.e., meta-data such as number of samples, quality control measures, and probe-to-gene conversion methods (e.g. GCRMA or PLIER in Affymetrix technology). These features are critical for the downstream gene ranking and interpretation, which is more desired nowadays. Also, they often fail to provide the correct dates of the experiments, and the associated protocol information. For example, some inaccuracies are a minor nuisance, like an experiment in SMD claiming to be performed on 11-16-1001 (instead of 11-16-2001), but others are more serious, like the problem mentioned in Figure 2 about caARRAY's default experiment dates. The issue of assigning an experiment date is unresolved in itself. Most microarray experimental results use arrays processed over a period of weeks, months, even years in some cases. When a data provider is expected to provide that field, they often just enter the publication date of the final paper. This is completely different from the timestamp of the data in the original arrays (e.g., the Affymetrix intensity data format contains a timestamp that the array was scanned). Until now, no microarray repository has attempted to extract and provide that data. This is one example of meta-data that the ArrayWiki import process automatically extracts and provides on the sample summary table of the main experiment page.

Another category of problem is the lack of evolvability of meta-data. That is the adjustment of meta-data based on the evolution of Microarray data standards. Before widespread adoption of the MGED Object Model (MGED-OM), microarray repository designers were left to invent their own labels for each column in their database. This led to a lack of agreement in what is appropriate to make a required field, and what meta-data (data labels) make the most sense to users. caARRAY is the only repository based entirely on MAGE-OM standards, but it is impossible to map experiments to their meta-data using the current search interface.

Finally, there is the issue of organization of the microarray data. Microarray experiments are often divided into classes, where only certain comparisons between certain sets of arrays make sense. ArrayExpress provides a visualization of the experiment MAGE-ML to help data consumers map the chip files to the right experimental conditions that they want to study. This process is not very intuitive, and requires the user to interpret the somewhat cryptic class names provided in the MAGE-ML. The ArrayWiki experiment page can be reorganized any number of ways to group samples. Descriptions of proper use of the data can also be added to guide data consumers to the right chip files.

Based on all the issues discussed above, we design and develop a Wiki repository, ArrayWiki, that can evolve meta-data standards at the rate of innovation driven by community involvement.

### Methodology and development of ArrayWiki

#### Wikis for collaborative knowledge curation

An important consideration when creating a biological data repository is the reuse of data standards accepted by the community. However, there is no effort underway to standardize human curation interactions with data repositories. Every repository still develops custom interfaces (usually web pages) for data access. For this reason, the most difficult part of hosting a repository is recruiting and maintaining interest in human curation. For instance, system interoperability efforts such as MAGE-OM [21], SBML [22], BioPAX [23,24], and caBIG [25] rely on XML for machine readability. On the other hand, the policy of many repositories of only allowing original data providers to modify their records adds to this problem. Technical experts might take the time to learn a specialized curation tool, but the wider community is unlikely to invest the time and effort [40]. For this reason, while data is becoming increasingly sharable, it is also becoming increasingly stale [26] because data owners are not always motivated to correct errors or respond to data consumer requests for changes.

Inspired by the spectacular success of the Wikipedia project , there have been efforts to compile biological knowledge in a Wiki format [27-29]. Also, there have even been suggestions that the whole of medical knowledge may one day be accessible through this format [30]. These efforts are largely motivated by the ease of use of Wikis and the ready availability of free and open source wiki software, such as MediaWiki . Wikis provide readable information for both humans and computer programs. In fact, recent publications have already shown that semantic web technologies such as automated annotation using Wikipedia pages have had some successes [31,41]. The dbpedia effort  is an open source project with the goal of automatically translating Wikipedia entries into the Resource Document Framework (RDF) format, which is a more recent and more flexible technology based on XML for Semantic Web. The Wiki paradigm is likely an important technology for data curation for semantic web research [32]. While a Wiki page does not yet provide a systematic and standard parsing structure that programmers expect from Extensible Markup Language (XML), the use of a smaller vocabulary of formatting syntax does improve the machine readability of its contents over that of unstructured general web contents.

Wikis hold their greatest promise in the dramatic advances over XML in human readability. Many users have the opportunity to modify Wiki data, and eventually consensus can be reached naturally. Media reports of the evolution of Wikipedia support this view. Many standards bodies (e.g. the SBML Consortium) already use Wiki software to accept community input before freezing a specification document. Wikis and efforts such as the Microarray Gene Expression Data (MGED) Society Ontology initiative [21] are mutually beneficial as the community seeks to homogenize language about microarray experiments. Future research may be able to integrate these two technologies [33].

#### Comparison of existing repository to wiki search features

The search mechanism that comes with the default installation of MediaWiki is a powerful way to mine available experimental data. All data available on the experiment summary page is treated equally by this search method. This new paradigm compares favorably with caARRAY, which uses a strict MAGE-OM but has not released any method to link experiments by protocols or platforms used for the purposes of search or download (as of version 1.4).

The GEO search tool allows for text searching in three different regions of their data structure: DataSets, Gene profiles and GEO accession records. However, the user must first discover just what each of these fields mean and most users are likely to attempt three separate searches to be sure of canvassing all available data.

SMD provides a very structured search interface. The user may specify only four pieces of meta-data: organism, experimenter, category and subcategory. These may be limited by using multi-select boxes, but there is no mechanism to search for fragments of keywords.

#### ArrayWiki design and development

In view of the limitations of repositories closed to community maintenance and the valuable features of Wiki knowledge repositories, we have developed ArrayWiki to host microarray data in an open environment, modifiable by any user. The ultimate goal of ArrayWiki is to unite data in all other repositories, while providing the most current analysis and the most detailed raw data. The following examples of ArrayWiki experiment pages and meta-data were all generated using a dataset that is unique to the ArrayWiki repository. It was originally intended to be distributed through GEO and is finally available to the public in this enhanced form. There are two experiments in this dataset, both related to renal cancer [34,35]. One experiment contains 24 hybridizations performed on fixed tissue with the Affymetrix X3P GeneChip. The other experiment contains 38 hybridizations using the Affymetrix HG-Focus GeneChip.

ArrayWiki pages are initialized programmatically by accessing APIs of GEO and ArrayExpress, or manually when an experiment does not exist in any repository yet. The current version contains over 500 experiments imported by GEO API. Quality control processes are still being run on these experiments to complete the import. A local database listing of all imported experiments ensures that existing pages are not overwritten each time the import process runs. A PHP class called Snoopy allows the import program to manipulate Wiki pages using HTTP POST, mimicking the process by which human users add contents. This is better than direct insertion into the database because it preserves the page history and the update tracking system.

ArrayWiki makes use of many useful add-ons to the MediaWiki software to enhance security and interoperability of data. One of these add-ons is the Captcha graphic for reducing automated spam generation. This feature requires the user to type a nonsensical word displayed in an image file whenever they add external links to a wiki page. Another add-on is the email image convertor. Contact emails are displayed as images in ArrayWiki to prevent mass harvesting of emails by automated scripts. Finally, custom scripts have been added to convert BioPNG files into the Affymetrix version 4 binary format CEL files. These files are made temporarily available for download by clicking the link and later deleted to preserve file system space.

In addition, the import process accesses raw data files and converts them to the BioPNG format (discussed later). This efficient storage method allows our system to support a greater data load and to make more efficient use of network bandwidth for downloads. This format offers greater protection against viruses than ZIP files (which may contain embedded executables). Finally, we have developed rules for determining when two datasets are duplicate records of the same experiments, in order to reduce overlap. Static links to source database records are provided where possible. Over time, we expect users to contribute more detailed data from past experiments based on emailed requests from the community.

ArrayWiki runs a number of automated quality control processes during import and all results are stored on the page. It is recommended that users download "clean" data when available. The import program uses a standard table format for all meta-data to improve machine readability. ArrayWiki also uses templates (in the style of Wikipedia) that allow Semantic Agents like dbpedia to better interpret structured data. Over time, ArrayWiki may prove to be a useful tool for community concensus-based development of data specification and curation standards.

**Figure 4:**
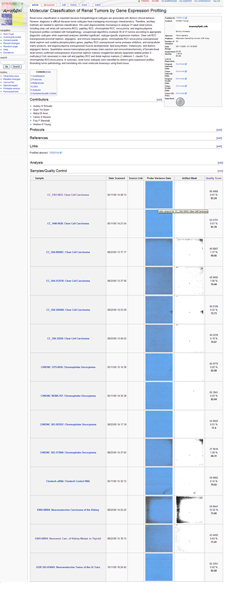
**A sample page in ArrayWiki showing experiment summary and detail**. The data in this experiment page was taken from [34, 35].

Figure 4 shows an example of an ArrayWiki experiment page containing 14 hybridizations. The ArrayWiki interface resolves many of the problems with community involvement. While it is not necessary that experimental data be submitted directly to ArrayWiki, the interface is simple and easy to use. The users can copy and paste the source code for a closely related experiment record and make only the necessary changes to describe a new experiment. This feature saves data contributor time, and is a great way to ensure that standard formatting is propagated.

#### Quality control

Quality control of data in ArrayWiki is provided by caCorrect [36]. This automated process generates key quality control meta-data whenever Affymetrix DAT or CEL files are detected in an experiment. This meta-data includes a heatmap of probe variance, a binary mask indicating which probes contain data of low confidence, a quality score for each chip, and an overall quality score for the whole experiment. The methods for generating this data are described in the original paper [36].

caCorrect can also provide two key value-added services. It can produce median-replaced probe data files (where untrustworthy data is replaced by the median probe value of chips in the same biological class) and GCRMA and PLIER gene expression calculations based on the clean data. Experiments are underway to demonstrate the importance of using microarray data cleaned with modern methods. ArrayWiki can save data consumers the time required to perform manual quality control steps on data downloaded from traditional repositories.

This data is so large that it would represent a significant burden on our server in its native format (uncompressed text or binary files). For this reason, all data is stored in BioPNG format (discussed below).

#### Compressed data storage

The BioPNG algorithm was developed to allow ArrayWiki to scale up faster while requiring less resources. Compression of laser scanner microarray data has been addressed by Luo and Lonardi [37]. The authors stress the importance of lossless compression and compare compression results of JPEG-LS, JPEG 2000, PNG, and TIFF image formats. They recommend JPEG 2000 but concede that this format lacks common browser support on the web. They also suggest (but don't implement) separating header info, foreground, and background pixels. As a trade-off between good compression and ready viewing of data, we have found PNG to be the most convenient.

**Figure 5:**
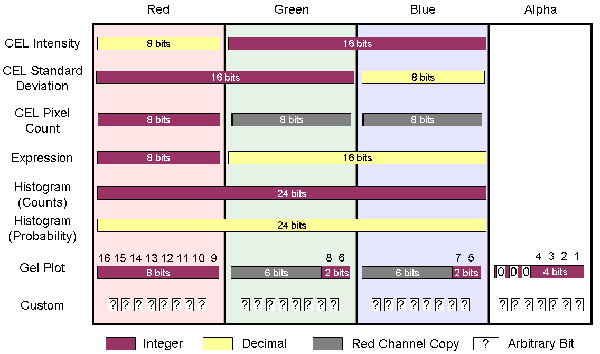
**Illustration of the BioPNG algorithm**. BioPNG specifies encoding methods for many types of numerical formats common to Affymetrix microarray experiments into a PNG file. Note that while Intensity and Standard Deviation data have the same storage requirements, different color channels were assigned to each part so that the files are immediately recognizable when viewed and one data file will not be mistaken for the other. Affymetrix CEL intensity data only contains one digit of precision in the decimal. In the case of normalized values, some precision may be lost in the conversion, but it is guaranteed to be less than 10^-4^. The purpose of the custom line is to show that the BioPNG API supports arbitrary definitions of color encoding, so that many more data types may be defined and be distinguishable from each other when viewing the images. The Gel Plot is just one example of a complex encoding scheme.

**Figure 6:**
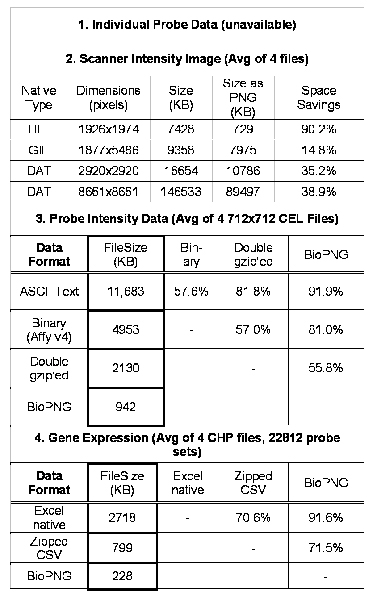
**Microarray data storage formats and relative compression ratios**. The numbering of the formats corresponds to that in Figure 1. Individual probe data is impossible to measure with current scanning technologies, but this is likely to change with technological advances. As the space savings gained using more complex compression become greater, the coding effort and computational expense to extract specific data points out of the files become greater. Retrieving a small set of intensity values from BioPNG is much faster because only those selected pixel values need to be converted.

BioPNG works by first splitting the numerical formats into coarse-grained and fine-grained bins (see Figure 5), and then making use of higher-order filters available in the PNG specification to model the data and store only the errors in the model. Affymetrix microarray data has many non-Gaussian correlations in the data that can be exploited for the purposes of compression. Our research has shown that different microarray platforms can differ significantly in the entropy (in the information theoretical sense of the term) of the data. We have calculated the first-order entropy of the HG_U95Av2 platform, containing 409600 intensity values, to be 10.1613 bits based on the samples we've processed. This means that an optimal first order compression algorithm should create files of average size 520.25 kilobytes (kB). By comparison, BioPNG compresses this data into files of size 767.42 kB (see Figure 6). Including more chips in the calculation of entropy will certainly raise this estimate, as not all of the available intensity symbols were used in our study. Still, our results show that BioPNG compression performs better than any custom first order compression algorithm, while still providing good portability and visualization.

Most repositories provide gene expression and probe intensity data in a zipped format. This can be problematic when attached to emails and may be infected with viruses by anonymous sources when shared on the web. ArrayWiki makes use of a novel algorithm that provides compression in a web-friendly format (generally considered virus-proof) and also allows for simple and extremely useful visualization of the underlying data. Our method, called BioPNG, encodes Affymetrix probe data as indicated in Figure 5(A). This method provides 12.4 times compression over ASCII text file storage and 2.26 times compression over GEO's method of zipping each binary Affy file individually and then zipping all of the files again into one file.

BioPNG's level of compression comes at small performance expense and no loss of data from the most important probe intensity measurement. What is lost is the data stored in the file header. These are automatically transferred into the experiment metadata in ArrayWiki. Spot standard deviation (STDV) and number of pixels read from the spot (NPIXELS) are stored in separate PNG files at high space savings. From our experience, this data is largely predictable and is of little interest beyond what is already detectable from the algorithm parameters.

All of the methods discussed in this section are readily transferable from cDNA microarrays to tissue, protein, and transfection array data. Work is underway to apply BioPNG compression to Illumina BeadChip arrays.

#### Visualization of data distributions, data errors, and quality problems

**Figure 7:**
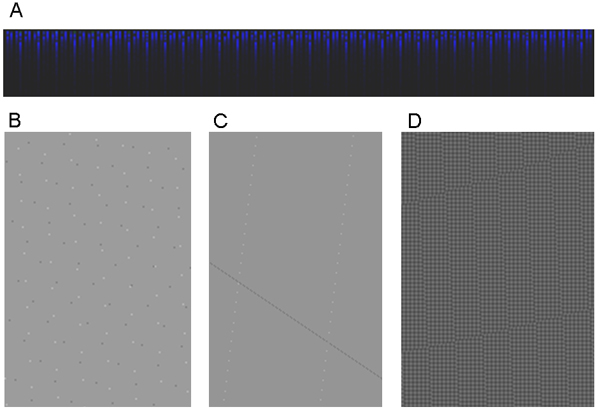
**Other Examples of BioPNG Histogram Count File**. (A) The BioPNG Histogram Count File can illustrate patterns in the frequency of different data values, another indicator of corrupt data. This example shows a periodic effect caused by lack of decimal values in higher intensity values that is not apparent in the lower intensity values. This is not necessarily a sign of data corruption, but may be related to rounding in the algorithm that converts DAT files to CEL files. (B) The BioPNG Pixel Count File can illustrate patterns in how the DAT to CEL conversion algorithm determines the number of pixels to use in the intensity calculation. Variations in this calculation are systematic and seem to correspond to very small differences in the orientation of the chip in the tray where it is read by the laser. Lighter pixels represent spots where more pixels being used in the calculation (and less being thrown out) while darker pixels represent fewer pixels bing used. Work is underway to determine if certain patterns are correlated to noisy, low-quality chip results. (These files have been cropped and brightened to make the patterns more visible in printed form.)

The BioPNG data format provides additional features for ensuring data quality in addition to providing compression and protection from cyber attacks. Recent improvements to the ArrayWiki import process provide a complete histogram of the original and the clean intensity data. This histogram stores the counts for all 490,000 possible values for intensity measurements in unprocessed CEL files, and the corresponding counts after the artifact removal process (see Figure 7A). Viewing this file can indicate data problems if single values are strangely over-represented or if an unexpected periodicity is observed in the data. Another histogram image stores the probability density function for each of these values, which is simply the hit counts normalized so they sum to 1. These images are useful in calculating the first- and second-order entropy of different microarray platforms.

**Figure 8:**
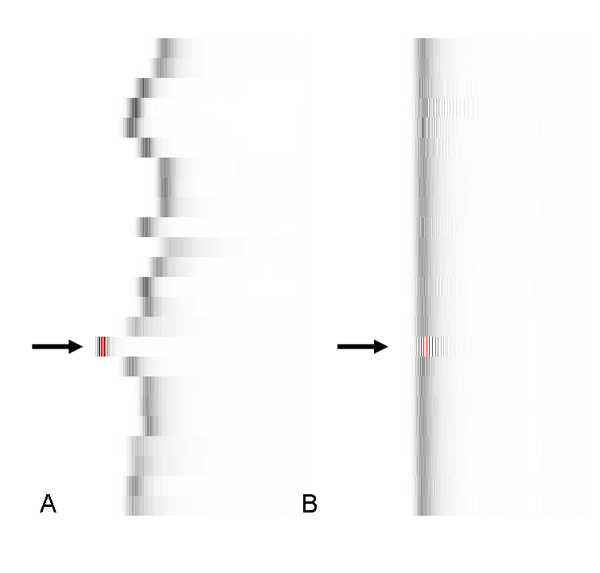
**The BioPNG Gel Plot**. (A) The gel plot of the original scanned data for the Renal Cell Carcinoma Affymetrix X3P GeneChip. The arrow indicates a chip with a corrupted file format, causing the intensity values to be read incorrectly. The Bioconductor program that parsed the chips did not catch this error, but it became clear after visualizing the intensity distributions. (B) The gel plot of the normalized and artifact-replaced data output by caCORRECT. These distributions are clearly well-aligned, but the same data parsing problem persisted without causing any post-processing algorithms to generate errors.

The Gel Plot visualization is another application of the BioPNG format to the data quality problem (see the example in Figure 8). The Gel Plot is created by first converting the intensity values into the log10 space, and then binning the values into 600 bins. Like other BioPNG formats, the decompression method can perfectly reconstruct the log10 intensity counts used to create the file. The storage of these values is unique compared to the other types of data. This is due to the objective of keeping a grayscale appearance, while working within the limitations of the library that the PHP programming language uses to create PNG Files. Using 16 bits for each color channel is supported in the PNG specification, but not in the PHP library. The Gel Plot data requires only 14 bits to store the highest possible count value (barring corrupt data), so the storage format uses each of the 8 bits of the "white" (rgb channels matching) and 6 bits of the alpha (or transparency) channel alternately. This simulates a 16-bit grayscale image, which produces more satisfactory results than a true 16-bit PNG because most browsers will not display all 65,536 available colors in a true 16-bit grayscale image.

Quality problems may arise in the algorithm that converts the intensity values read by the scanner (available in the DAT file) into the values reported in the CEL file. The final effect on reported gene expression has not been quantified, but the extent of the potential problems is visualized by the BioPNG-formatted NPIXEL file. This column of data in a CEL file indicates how many pixels were used in the calculation of the intensity. This number generally ranges from 12 to 36. Systematic patterns (as seen in Figure 7B–D) may indicate misalignment between the track of the laser and the grid of the microarray spots. In future work, our aim is to show whether extreme misalignment may result in low-quality gene expression calculations.

## Discussion

ArrayWiki is not limited to the meta-data and quality control information presented here. An example of additional useful information about microarray experiments is discussed by Torrance et. al. [38], who have developed methods to visualize and quantify co-occurrence between microarray outlier probes and those probe sets found to be biologically significant. No existing microarray repository provides any means to associate follow-on studies (whether positive or negative) with existing experiments. Additionally, the FDA MAQC project suggests new visualization methods (e.g., volcano plots) for determining bias in microarray experiments. These follow-on studies are critical for future data consumers to determine the importance and trustworthiness of microarray experiments and results. The current version of ArrayWiki does not take full advantage of the Wiki-based, programmatically managed architecture. ArrayWiki is based on open-source software, so any number of custom scripts and changes to the original MediaWiki configurations are allowed and encouraged. There is also an active community of users providing MediaWiki extensions for adding functionality like automated detection of cross-references and creation of links between technical words and their corresponding Wikipedia entries.

ArrayWiki could also benefit from the development of Meta-Pages. In Wikipedia, this means pages created not as encyclopedia articles (or experiment summaries), but strictly for adding an additional level of organization to the site. In the case of ArrayWiki, these Meta-Pages may present a summary of available data in the format of pie charts like those in Figure 1. They might also include a list of the Top 100 most popular experiments. The potential for Meta-Pages is limited only by the imagination of the community. Future work includes comparing the compression ratio of BioPNG to the second order entropy of microarray experiments to demonstrate how much storage might be saved by implementing more sophisticated but less portable compression algorithms.

## Conclusion

ArrayWiki provides information about data quality (not found in other repositories) to help guide users toward the best data for reproducibility. ArrayWiki uses BioPNG which is a safe and compact information transport format allowing visualization of many new features of microarray data.  ArrayWiki provides a shared space for curation of microarray experiment data and deposition of meta-analysis results. Similar to Wikipedia, with simple formatting skills, users can organize, update and contribute new analysis and meta-data to ArrayWiki over time to keep the information up-to-date. Ultimately, with continued accumulation of user curation and validation, ArrayWiki helps to improve the overall reliability of microarray data.

## Competing interests

The authors declare that they have no competing interests.

## Authors' contributions

T.H.S. conceived the ideas of ArrayWiki and BioPNG that enable the research effort in comparing existing microarray repositories, and designed the data model for tracking the import process and for integrating quality control modules. J.T.T. contributed to the majority of the database overlap statistics and suggestions for community contributions. H.L. wrote import scripts for ArrayWiki and contributed to the final page layout. M.D.W. is the faculty advisor directing the whole project and final publication.
